# Reduction in anxiety and depression symptoms one year after knee replacement: a register-based cohort study of 403 patients

**DOI:** 10.1007/s00590-020-02860-7

**Published:** 2021-01-11

**Authors:** Aamir Mahdi, Maria Hälleberg-Nyman, Per Wretenberg

**Affiliations:** 1 Department of Orthopaedics, Örebro County Council, Örebro, Sweden; 2 Faculty of Medicine and Health, School of Medical Sciences, Örebro University, Örebro, Sweden; 3 Faculty of Medicine and Health, School of Health Sciences, Örebro University, Örebro, Sweden

**Keywords:** Anxiety, Depression, Hospital anxiety and depression scale, Patients-related outcome measures, Total knee arthroplasty

## Abstract

**Background:**

Anxiety and depression are associated with patient dissatisfaction after total knee arthroplasty (TKA). Understanding whether preoperative knee-related symptoms could be a cause of anxiety and depression might help prevent unnecessary delay of surgery for this group of patients. We investigated changes in prevalence of anxiety and depression symptoms one year after TKA, and compared demographic data between patients with and without anxiety and depression symptoms preoperatively.

**Methods:**

This was a prospective cohort study of 403 patients scheduled for TKA. Data on patient-related outcome measures and the prevalence of anxiety and depression symptoms were collected preoperatively and one year postoperatively. Before–after differences in anxiety/depression prevalence were compared with a chi-square test, and differences in demographic data between the groups with and without anxiety and/or depression symptoms were compared with an independent t test.

**Results:**

Among the 15% of patients with anxiety symptoms before surgery, 59% had improved in these symptoms one year after surgery; while among the 10% with depression symptoms before surgery, 60% had improved one year after surgery. Patients with preoperative anxiety and/or depression were younger, and had higher body mass index, lower general quality of life (EQ-5D-3L), higher pain scores (visual analog scale), and lower knee-related (KOOS) scores on all subscales except sport.

**Conclusion:**

Presurgical symptoms of anxiety and depression seem to be partly caused by knee symptoms. Understanding of this issue would offer better strategies to prevent unnecessary delay of surgery in this group of patients.

## Background

Osteoarthritis is a common and disabling disease of the knee joint [1–3]. Total knee arthroplasty (TKA) is effective in improving pain, function, and quality of life for patients with knee osteoarthritis [4–6], but about a fifth of patients are not satisfied after the operation [7–9]. Preoperative depression and anxiety are predictors for dissatisfaction after TKA [10–12]. Overall, a fifth of patients with osteoarthritis experience anxiety and/or depression [13]. However, the annual report from Swedish knee arthroplasty registry (SKAR) in the last two years showed that about 35% of Swedish patients who underwent TKA were reported anxiety symptoms before surgery[14, 15].

Anxiety is a disorder in which the feeling of fear is persistent and overwhelming in relation to a normal situation [16, 17]. Its prevalence in a general population can be as high as 33% [16, 18]. In contrast, only 4–8% of people have a clinical diagnosis of depression. Symptoms of depression are much more frequent than this, but only about a third of patients with depression symptoms require treatment [19].

Previous literature recommends that patients’ anxiety and depression should be assessed and managed before surgery in order to decrease the dissatisfaction rate [10, 20, 21]. However, there is little knowledge about whether the symptoms of depression and anxiety are a result of symptoms and disabilities caused by the knee osteoarthritis. One study showed that increasing disability and poorer quality of life are reasons for higher anxiety prevalence among elderly people [22]. Others found that depression improved after hip/knee replacement surgery due to a reduction in pain [23], and that depression and anxiety symptoms improved 6 months after surgery [24].

The main aim of this study was therefore to investigate changes in the prevalence of anxiety and depression one year after primary TKA. Understanding of this issue might lead to a better preoperative assessment of patients scheduled for TKA. The second aim was to compare preoperative demographic data between patients who did and did not have anxiety and/or depression symptoms, in order to help build up a preoperative profile of patients with these symptoms.

## Methods

### Patient selection

We performed a prospective cohort study with a consecutive sample of patients scheduled for knee replacement surgery between April 2016 and April 2018 at three hospitals in a county in central Sweden, one university hospital and two local hospitals, serving in total approximately 300,000 people. The surgeons used a standard paramedian approach. Each orthopedic surgeon performed between 25 and 75 primary TKAs per year. The inclusion criteria were having knee osteoarthritis and being scheduled for primary knee replacement surgery at one of the three hospitals. The exclusion criteria were being scheduled for a revision of knee arthroplasty or a unicompartmental knee arthroplasty, or having a diagnosis other than osteoarthritis.

Patient-reported outcome measures (PROMs) were assessed with a questionnaire used by the SKAR. This registry collects individual-based data on patients and surgeries. SKAR provided us with the PROMs questionnaire used annually by the registry, software for entering and calculating scores on the Hospital Anxiety and Depression Scale (HADS), and access to SKAR’s data program for entering PROMs and HADS data. This access concerned only our patients and was restricted to the common database. Thus, we took advantage of SKAR routines in terms of using a well-validated questionnaire and high-quality software which protects the patient’s identity.

### Outcome measures

The patients were asked to fill out a PROMs questionnaire at two time points: before surgery and one year after surgery. The questionnaire included the Knee Injury and Osteoarthritis Outcome Score (KOOS), a visual analog scale (VAS) for measuring pain, a general quality of life questionnaire (EQ-5D-3L), and HADS.

KOOS is a knee-specific questionnaire comprising five subscales: pain (9 items), other symptoms (7 items), activities of daily living (17 items), sport and recreation function (5 items), and knee-related quality of life (4 items). It takes about 10 min to answer the questions [25, 26].

A VAS is the commonest and easiest pain rating scale. The patient rates their pain intensity from 0 to 100, with 0 representing no pain and 100 representing the highest pain intensity. It takes less than a minute to complete [27]. SKAR uses the VAS to measure both the patient’s current pain and the patient’s expectation of what their pain would be after surgery.

EQ-5D-3L is a five-dimensional three-level generic instrument covering mobility, self-care, usual activities, pain/discomfort, and anxiety/depression. It is well-known and takes less than a minute to complete [28].

HADS is a simple questionnaire which takes 2–5 min to complete. It consists of 7 items for anxiety and 7 items for depression. The minimum score is 0 and the maximum is 21 for each of the sub-scores. Patients are considered to have anxiety or depression symptoms if their HADS-Anxiety (HADS-A) or HADS-Depression (HADS-D) score, respectively, is more than 7 [29].

All of these PROMs questionnaires have been psychometrically validated [28, 30].

### Statistics

A chi-square test was used to compare the two groups with/without anxiety before and one year after surgery. The dependent variable was anxiety symptoms pre-surgery, the independent variable was TKA, and the control variable was symptoms one year postoperatively.

An independent t test was used to compare demographic data between the two groups with and without anxiety before surgery. Similar statistical tests were performed for depression symptoms.

McNemar test was used for pre- and postoperative comparisons of anxiety and depression. Odds ratios were calculated, as were 95% confidence intervals (CIs) when appropriate. Differences were regarded as significant when the p value was less than 0.05. Version 25 of the SPSS software package (IBM, SPSS Inc., Chicago, Illinois, USA) was used for the quantitative analyses.

The power of the study was calculated on the basis of previous studies [23, 24] and a sample size calculator [31]. We expected that 10–20% of patients (*n* = 30–60) would have symptoms of anxiety and/or depression, and hence estimated that enrolling about 300 patients would be enough to produce statistically significant differences with a power of 0.8 and alpha of 0.05. However, we enrolled 403 patients to compensate for eventual attrition.

The effect size was calculated by measuring the eta-squared value using SPSS with both cross-table comparison and univariate analysis of variance. Eta-squared define the effect size as small, medium and large when its value is 0.01, 0.06 and 0.14, respectively [32, 33].We considered the large effect size to be clinically important.

## Results

**Fig. 1:**
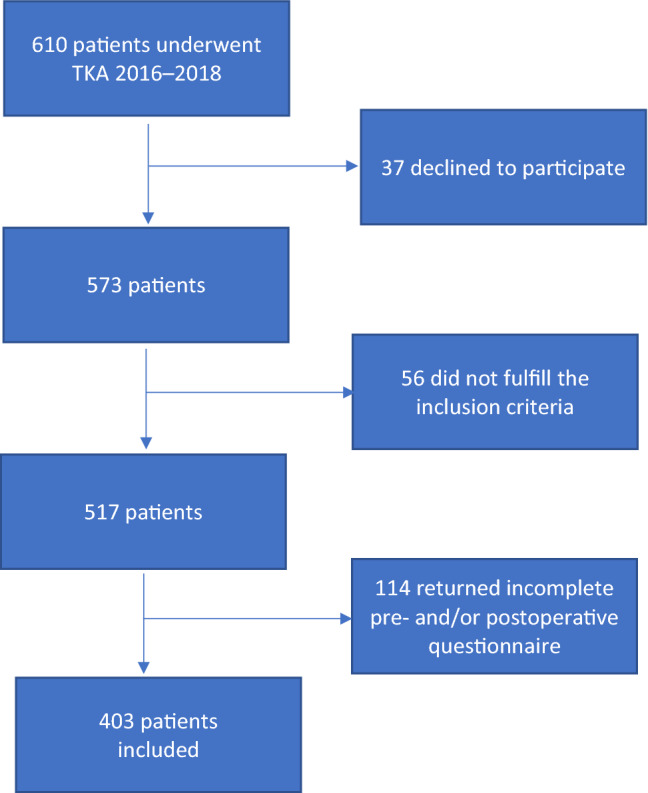
Flowchart of patient inclusion in the study

Of the 610 patients who underwent surgery between April 2016 and April 2018, 207 were excluded. Of them, due to declining participation (*n* = 37), unicompartmental knee arthroplasty (*n* = 22), revision surgery (*n* = 12), diagnosis other than primary osteoarthritis (*n* = 22), and incomplete data (*n *= 114), leaving 403 included in the study (94% response rate) (Fig. 1).

Reasons for incomplete data were:

1. At the beginning of the study the nurses had not yet got into the routine of distributing the questionnaire, which caused them to miss some patients
2. Patients delaying their answers for more than 3 months
3. Incomplete answering of the questionnaire
4. Patients not returning the preoperative or postoperative questionnaire despite reminders
5. Death

Missing data were distributed randomly, and did not affect a specific group or hospital. Demographics of the patients with incomplete data are shown in Table 1.

**Fig. 2:**
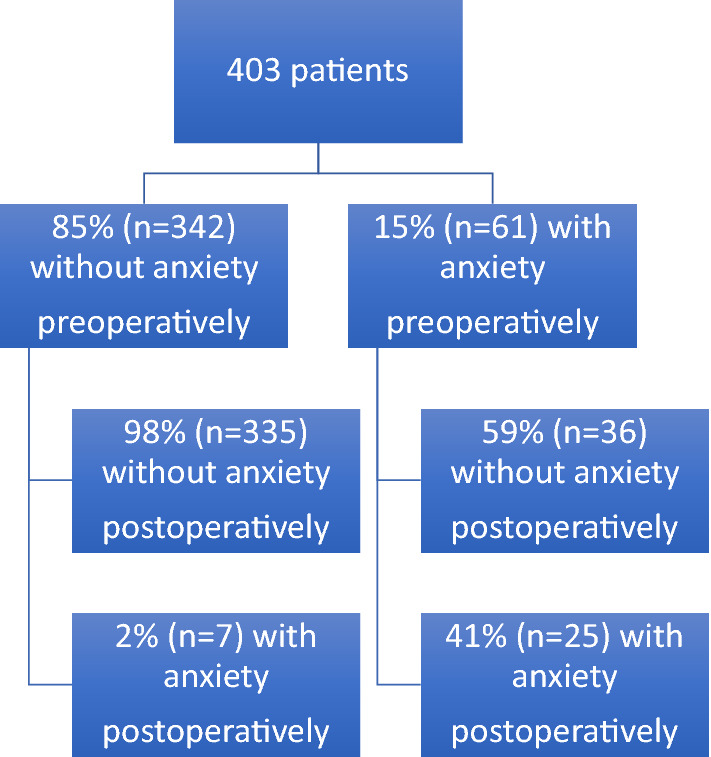
Improvement in anxiety symptoms one year after total knee arthroplasty

Of the 403 patients with complete data, 15% (*n* = 61) reported significant anxiety symptoms (HADS-A ≥ 8) before TKA, while the remaining 85% (*n* = 342) were regarded as not having anxiety symptoms (HADS-A < 8). One year after surgery, the group with anxiety showed a significant reduction in HADS-A score, with 59% of the patients in this group now having normal scores (*n* = 36; *p* < 0.001). The remaining 41% (*n* = 25) continued to report anxiety one year after surgery. Thus, the odds ratio for being anxiety-free one year after surgery was 1.6 (95% CI: 1.3–2.0; *p* < 0.001). Further analysis of the group without preoperative anxiety showed that 98% (*n* = 335) of them still reported no anxiety symptoms one year after surgery, while symptoms had developed in 2% (*n* = 7). Thus, the odds ratio for having anxiety symptoms one year after surgery was 0.05 (95% CI: 0.02–0.11; *p* < 0.001) (Fig. 2).

**Fig. 3:**
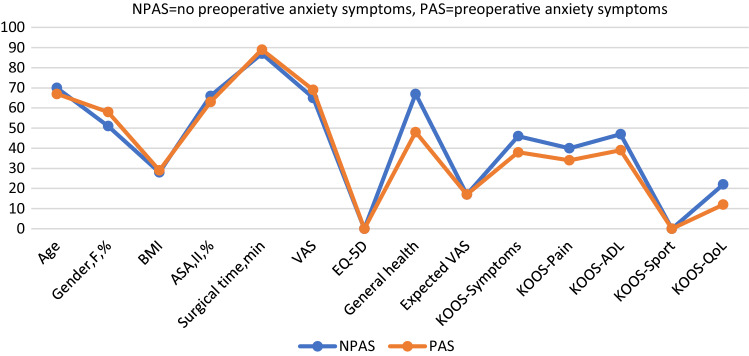
Comparison of preoperative anxiety and non-anxiety groups

Further demographic analysis of both groups preoperatively showed that the patients with preoperative anxiety symptoms tended to be younger, and to have higher body mass index (BMI), lower EQ-5D index, lower general health, and lower scores on the KOOS subscales for symptoms, pain, activities of daily living, and knee-related quality of life. All these differences were statistically significant (Table 2; Fig. 3). Gender, American Society of Anesthesiologists (ASA) class, surgical time, preoperative VAS, expected VAS, and preoperative KOOS sport/recreation did not differ significantly between the groups (Table 2; Fig. 3).

**Fig. 4:**
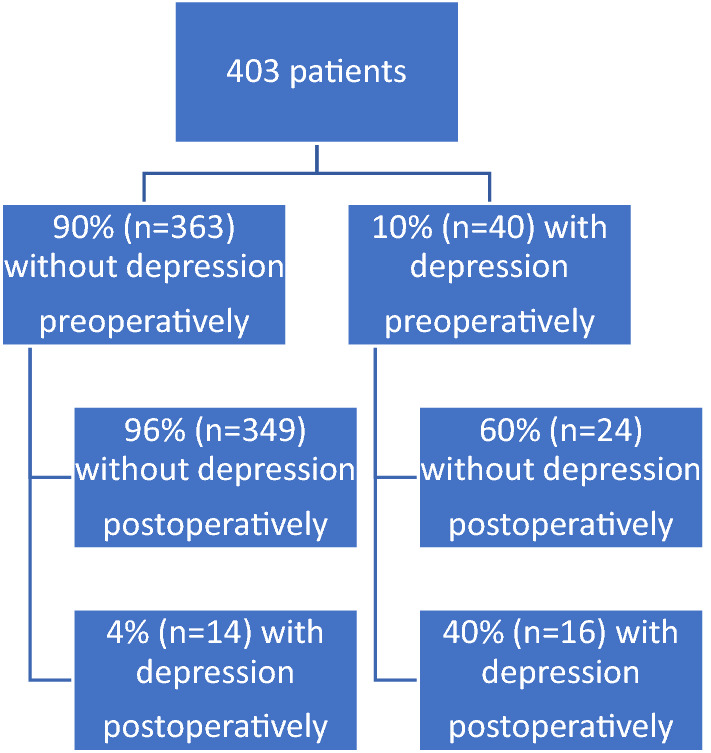
Improvement in depression symptoms one year after total knee arthroplasty

Of the 403 patients, 10% (*n* = 40) reported significant depression symptoms (HADS-D ≥ 8) before TKA, while the remaining 90% (*n* = 363) were regarded as not having depression (HADS-D < 8). One year after surgery, the group with depression showed a statistically significant reduction in HADS-D score, with 60% of the patients in this group now having normal scores (*n* = 24; *p* < 0.001) The remaining 40% (*n* = 16) continued to report depression one year after surgery. Thus, the odds ratio for being depression-free one year after surgery was 1.6 (95% CI: 1.2–2.0; *p* < 0.001). Further analysis of the group without preoperative depression showed that 96% (*n* = 349) of them still reported no depression symptoms one year after surgery, while symptoms had developed in 4% (*n* = 14). Thus, the odds ratio for having depression symptoms one year after surgery was 0.09 (95% CI: 0.05–0.18; *p* < 0.001) (Fig. 4).

**Fig. 5:**
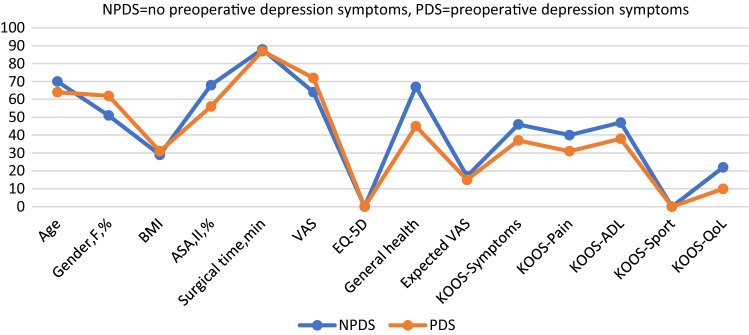
Comparison of preoperative depression and non-depression groups

Further demographic analysis of both groups preoperatively showed that the patients with preoperative depression symptoms tended to be younger, and to have higher BMI, higher preoperative VAS, lower preoperative EQ-5D index, lower general health, and lower scores on the KOOS subscales for symptoms, pain, activities of daily living, and knee-related quality of life. All these differences were statistically significant (Table 3; Fig. 5). Gender, ASA class, surgical time, expected VAS, and preoperative KOOS sport/recreation did not differ significantly between the groups (Table 3; Fig. 5).

**Fig. 6:**
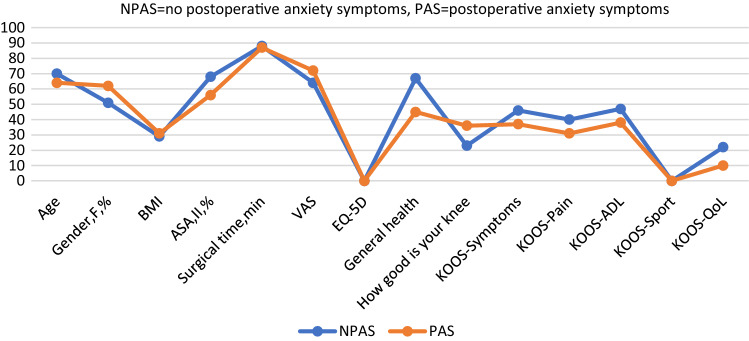
Comparison between groups with and without postoperative anxiety symptoms

**Fig. 7:**
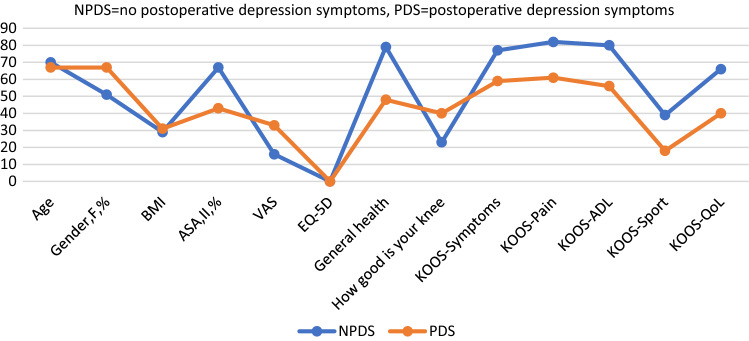
Comparison between groups with and without postoperative depression symptoms

Patients who showed anxiety symptoms (*n* = 32) and/or depression symptoms (*n* = 30) one year after surgery differed from patients without these symptoms in terms of being younger and more likely to be female, having higher BMI, and reporting higher postoperative pain, lower general health, increased comorbidity, generally less satisfaction with their knees, and lower KOOS scores on all subscales (Tables 4, 5; Figs. 6, 7).

There were seven patients who did not report anxiety symptoms before surgery but they developed such symptoms one year after surgery. Similarly, there were also 14 patients who did not report depression symptoms before surgery but they reported such symptoms one year after surgery. The changes were statistically significant regarding anxiety but not depression (*p* < 0.0001 and *p* = 0.143, respectively).

Clinical significance was measured by calculating the effect size (eta-squared value) of the reduction in anxiety and depression symptoms. Eta-squared was 0.26 (*p* < 0.001) for anxiety and 0.17 (*p* < 0.0001) for depression. Both values were considered a large effect size and considered being clinically relevant.

## Discussion

This study examined changes in the prevalence of anxiety and depression symptoms between two time points, before and one year after a total knee arthroplasty in patients with knee osteoarthritis. We showed a significant reduction in anxiety and depression symptoms one year after surgery in patients with such symptoms prior to surgery.

Many previous studies have shown the negative impact of anxiety on the outcome after TKA [7, 8, 20, 21, 34]. This could be used as an argument not to operate on patients with anxiety or depression symptoms, or at least to postpone surgery. However, it is not ethical to either totally exclude these patients from surgery or extend their suffering by postponing surgery. On the other hand, it is important to accurately assess the patient’s preoperative anxiety state in order to avoid patient dissatisfaction, which could be defined as poor results after surgery.

The prevalence of anxiety in a general population varies in the literature, but is estimated to be as high as 33% [16, 18]. It is difficult to say whether the symptoms of anxiety found in our study were related to knee symptoms or were part of the general population prevalence.

The significant reduction in anxiety symptoms that we found one year after surgery might be important for knee surgeons to know when assessing patients preoperatively. Taking into account the improvement of anxiety symptoms in 59% of patients could decrease the discrimination against patients with anxiety symptoms. In addition, our description of the demographics of patients with preoperative anxiety symptoms might aid knee surgeons in clearly defining these patients preoperatively.

Our interpretation of the results is that many patients experienced anxiety/depression symptoms before surgery due to knee symptoms. This interpretation is supported by our further analysis of patients who continued to have anxiety and/or depression one year after surgery. The analysis revealed that these patients had more knee pain and lower KOOS subscale scores postoperatively compared to patients who did not show anxiety/depression symptoms one year after TKA. Our results are in line with previous studies showing the impact of knee symptoms on patients’ anxiety state [22, 24, 35].

The prevalence of depression symptoms before TKA in our data was 10%. A statistically significant 60% reduction in these symptoms one year after surgery might indicate the effect of knee symptom alleviation after surgery. This result is also in line with previous studies [23, 35]. Tarakji et al. (2018) showed a post-surgery improvement in depression symptoms, decrease in pain, and improvement in physical function, and interpreted this as an additional benefit of knee arthroplasty. We found that patients who described more depression symptoms before and after surgery had more knee pain and symptoms than patients who did not have depression symptoms.

In contrast, most earlier studies investigated the negative impact of depression on the outcome after knee arthroplasty [7, 10, 11, 36, 37]. Here, again, relying on these studies would lead to discriminating against a group of patients who would be helped by knee arthroplasty, and hence unnecessarily increasing their suffering by excluding or postponing them from surgery. As with anxiety, the demographic data we report here can provide a profile of patients with and without depression symptoms before surgery, which could help in correctly analyzing patients with preoperative depression symptoms.

A strength of this study was its prospective cohort design with large sample size. This allowed the calculation of prevalence and odds ratios for both groups with anxiety and depression symptoms before and after surgery. The results were not only statistically significant but also showed a clinically significant impact. The effect size of symptom reduction was considered to be large because the eta-squared value was larger than 0.14 [32, 33].

Another strength was that we included all patients with knee osteoarthritis who underwent TKA at three different hospitals. This served to minimize the risk of selection bias even though many cases were lost to follow-up.

The relatively large number of missing cases (*n* = 114, 18%) could be regarded as a weakness. However, it did not affect any one specific group, and it was distributed randomly among the patients. Further analysis of patients with incomplete data was performed. This showed that these patients tend to be younger, have higher BMI, more often Charnley class C and reported more presurgical anxiety symptoms. On the other hand, there were no significant differences regarding gender, ASA and presurgical depression symptoms.

Another limitation was the dichotomization of anxiety and depression before and after surgery, meaning that we did not take the severity of the depression and anxiety symptoms into account. Further classification of anxiety and depression symptoms into mild, moderate, and severe would have spread the patients into several subgroups, leading to a small number of patients in each subgroup, which in turn would have affected the statistical significance of the results. Further studies with larger sample size are needed in order to analyze the subgroups of anxiety and depression symptoms and clarify the role of symptom severity in relation to improvement of symptoms one year after TKA.

Another limitation was relating to the fact that both anxiety and especially depression symptoms are episodic and often recurrent with spontaneous worsening and improvement [38, 39]. Thus, we do not know the number of patients who improved spontaneously or as a result of improved knee symptoms. On the other hand, the same principle should apply in patients with spontaneous worsening of anxiety/depression symptoms. Moreover, comparison among the groups in this study showed that patients with anxiety or depression symptoms had significantly higher knee complaints whether before or after surgery. Moreover, the HADS score of below 7 is regarded in the literature as normal value regardless of the variations below this score [29].

## Conclusions

This study showed a significant reduction in anxiety and depression symptoms one year after TKA, which has clinical implications for daily orthopedic work. Our results can serve as a tool to support the beneficial effect of TKA in patients with knee arthrosis alongside anxiety or/and depression symptoms. Excluding this group of patients or postponing surgery would lead to more patient suffering as well as a higher economic burden on the community. Further studies with larger samples are needed to examine the subgroups of patients with anxiety and depression symptoms.

**Table 1:** Demographics of the patients with incomplete data compared with complete data

Characteristics	Incomplete data, *n* (total *n* = 114)	Complete data, n (total = 403)	*p* value
Gender, *n* (%)			
Female	65 (57)	211 (52)	0.38
Male	49 (43)	192 (48)	
Age, months	68	70	0.002
BMI, m	30.8	28.8	< 0.001
ASA, *n* (%)			
I	24 (21)	107 (26)	0.44
II	82 (72)	265 (66)	
III	8 (7)	31 (8)	
Charnley class, *n* (%)			
A	19 (17)	105 (26)	0.001
B1	17 (15)	104 (26)	
B2	20 (17)	60 (15)	
C	58 (51)	134 (33)	
PAS, *n* (%)			
No	72 (63)	342 (85)	0.004
Yes	27 (24)	61 (15)	
Missing	15 (13)		
PDS, *n* (%)			
No	86 (76)	363 (90)	0.35
Yes	13 (11)	40 (10)	
Missing	15 (13)		

*BMI* body mass index; *ASA* American society of anesthesiologists; *PAS* preoperative anxiety symptoms; *PDS* preoperative depression symptoms.

**Table 2:** Demographics and comparison of preoperative anxiety and non-anxiety groups

Variable	NPAS (*n* = 342)	PAS (*n* = 61)	Mean difference	95% CI of the difference		Significance (2-tailed)
				Lower	Upper	
Age (m)	70	67	3	1	5	0.002
Gender (%)						
Female	51	58				0.27
Male	49	42				
BMI (m)	28	29	− 1.1	− 2.3	− 0.06	0.036
ASA class (%)						
I	27	25				0.28
II	66	63				
III	7	12				
Surgical time (min)	87	89	− 3	− 8.5	3	0.34
Preop VAS (m)	65	69	− 4.1	− 8.7	0.34	0.07
Preop EQ-5D index (m)	0.49	0.30	0.18	0.11	0.26	0.00
Preop general health (m)	67	48	18.5	12.9	24.2	0.00
Expected VAS (m)	16.7	16.9	− 0.15	− 3.7	3.4	0.93
Preop KOOS symptoms (m)	46	38	7.7	3	12	0.001
Preop KOOS pain (m)	40	34	6	2.2	9.9	0.02
Preop KOOS ADL (m)	47	39	8	4	12	0.00
Preop KOOS sport (m)	10.3	8.7	2.4	− 0.8	5.7	0.14
Preop KOOS quality of life (m)	22	12	9.3	5.7	12.8	0.00

*NPAS* no preoperative anxiety symptoms; *PAS* preoperative anxiety symptoms; *CI* confidence interval; *m* mean; *BMI* body mass index; *ASA* American society of anesthesiologists; *preop* preoperative; *VAS* visual analog scale; *KOOS* knee injury and osteoarthritis outcome Score; *ADL* activities of daily living; *min* minutes.

**Table 3:** Demographics and comparison of preoperative depression and non-depression groups

Variable	NPDS (*n* = 363)	PDS (*n* = 40)	Mean difference	95% CI of the difference		Significance (2-tailed)
				Lower	Upper	
Age (m)	70	64	5.9	3.4	8.3	< 0.001
Gender (%)						
Female	51	62				0.09
Male	49	38				
BMI (m)	28.6	30.9	− 2.2	− 3.6	− 0.9	0.04
ASA class (%)						
I	25	34				0.20
II	68	56				
III	7	10				
Surgical time (min)	88	87	− 0.72	− 6.2	7.6	0.83
Preop VAS (m)	64	72	− 7.2	− 12.6	− 1.8	0.009
Preop EQ5D index (m)	0.48	0.28	0.19	0.10	0.29	< 0.001
Preop general health (m)	67	45	22	15	28	< 0.001
Expected VAS (m)	17	15.5	1.20	− 2.9	5.5	0.50
Preop KOOS symptoms (m)	46	37	8.9	3.3	14.6	< 0.001
Preop KOOS pain (m)	40	31	9	4	13	< 0.001
Preop KOOS ADL (m)	47	38	9	4	13	< 0.001
Preop KOOS sport (m)	10.2	7.75	2.45	− 1.54	6.44	0.22
Preop KOOS quality of life (m)	21.9	10.4	11.5	7.5	15.4	< 0.001

*NPDS* no preoperative depression symptoms; *PDS* preoperative depression symptoms; *CI* confidence interval; *m* mean; *BMI* body mass index; *ASA* American Society of Anesthesiologists; *preop* preoperative; *VAS* visual analog scale; *KOOS* Knee Injury and Osteoarthritis Outcome Score; *ADL* activities of daily living; *min* minutes

**Table 4:** Comparison of variables between groups with and without postoperative anxiety symptoms

Variable	NPAS (*n* = 371)	PAS (*n* = 32)	Mean difference	95% CI of the difference		Significance (2-tailed)
				Lower	Upper	
Age (m)	70	67	3	1	5	0.033
Gender (%)						
Female	50	78				0.002
Male	50	22				
BMI (m)	28	30	− 1.8	− 3.3	− 0.3	0.019
ASA class (%)						
I	26	28				0.89
II	66	62				
III	7.5	9				
Postop VAS (m)	16	32	− 15.5	− 22	− 8	< 0.001
Postop EQ5D index (m)	0.81	0.48	0.33	0.26	0.40	< 0.001
Postop general health (m)	78	51	27	20	34	< 0.001
How good is your knee	23	36	− 13	− 21	− 4.6	0.002
Postop KOOS symptoms (m)	77	62	14	8	21	< 0.001
Postop KOOS pain (m)	82	64	18	11	24	< 0.001
Postop KOOS ADL (m)	80	61	19	12.5	26	< 0.001
Postop KOOS sport (m)	38	22	16	7	26	0.001
Postop KOOS quality of life (m)	65.5	43	22	14	30	< 0.001

NPA*S* no postoperative anxiety symptoms; *PAS* postoperative anxiety symptoms; *CI* confidence interval; *m* mean; *BMI* body mass index; *ASA* American society of anesthesiologists; *postop* postoperative; *VAS* visual analog scale; *KOOS* knee injury and osteoarthritis outcome score; *ADL* activities of daily living; *min* minutes

**Table 5:** Comparison of variables between groups with and without postoperative depression symptoms

Variable	NPDS (*n* = 373)	PDS (*n* = 30)	Mean difference	95% CI of the difference		Significance (2-tailed)
				Lower	Upper	
Age (m)	70	67	3	0	6	0.05
Gender (%)						
Female	51	67				0.10
Male	49	33				
BMI (m)	28.7	30.7	− 2	− 3.6	− 0.5	0.01
ASA class (%)						
I	26	37				0.006
II	67	43				
III	7	20				
Postop VAS (m)	16	33	− 16	− 23	− 9	< 0.001
Postop EQ-5D (m)	0.81	0.41	0.39	0.32	0.46	< 0.001
Postop general health (m)	79	47.5	31	24	38	< 0.001
How good is your knee	23	40	− 17	− 26	− 8.5	< 0.001
Postop KOOS symptoms (m)	77	59	17.7	11	24	< 0.001
Postop KOOS pain (m)	82	61	21	14	27	< 0.001
Postop KOOS ADL (m)	80	56	24	17	31	< 0.001
Postop KOOS sport (m)	39	18	21	11	31	< 0.001
Postop KOOS quality of life (m)	66	40	25	17	34	< 0.001

*NPDS* no postoperative depression symptoms; *PDS* postoperative depression symptoms; *CI* confidence interval; *m* mean; *BMI* body mass index; *ASA* American Society of Anesthesiologists; *postop* postoperative; *VAS* visual analog scale; *KOOS* knee injury and osteoarthritis outcome score; *ADL* activities of daily living; *min* minutes

## Data Availability

The datasets used and/or analyzed during the current study are available from the corresponding author on reasonable request.

## Abbreviations

ASA: American Society of Anesthesiologists
BMI: Body Mass Index
CI: Confidence Interval
HADS: Hospital Anxiety and Depression Scale
KOOS: Knee injury and Osteoarthritis Outcome Score
PROMS: Patient-Related Outcome Measures
SKAR: Swedish Knee Arthroplasty Register
TKA: Total Knee Arthroplasty
VAS: Visual Analog Scale
